# Prescribing Aspirin for Preeclampsia Prevention in Pregnant Women During COVID-19: Should or Shouldn’t?

**DOI:** 10.22037/ijpr.2021.115076.15183

**Published:** 2021

**Authors:** Laleh Eslamian, Marjan Ahmadi, Mahnaz Ahmadi

**Affiliations:** a *Department of Obstetrics and Gynecology, Perinatology Unit, Shariati Hospital, Tehran University of Medical Sciences, Tehran, Iran. *; b *Department of Pharmaceutics, School of Pharmacy, Shahid Beheshti University of Medical Sciences, Tehran, Iran.*

One of the most common complications during pregnancy is preeclampsia, a multisystemic disorder that results in high blood pressure and multiple organ damage. It usually begins after 20 weeks of gestation. It is estimated that 2-8% of pregnant women experience eclampsia worldwide. Each year more than 287,000 women die due to pregnancy-related diseases, and eclampsia accounts for 10-15% of them (1). It is suggested that prescribing low-dose aspirin prevents preeclampsia in 60-90% of pregnant women (2).

COVID-19 pandemic began in December 2019 as an acute respiratory disease with severe morbidities and high mortality rates. Still, we know little about COVID-19 in pregnancy. The studies demonstrate that pregnant women are at higher risk of experiencing more severe disease (3).

There is concern about aspirin administration, which could have side effects on patients with respiratory illnesses such as COVID-19. The American College of Obstetricians and Gynecologists (ACOG), International Federation of Gynaecology and Obstetrics (FIGO), Society for Maternal-Fetal Medicine (SFMF), and Society of Obstetricians and Gynaecologists of Canada (SOGC) guidelines stated that during the pandemic, low-dose aspirin could be prescribed to pregnant women as clinically indicated. For the patients infected with COVID-19 or at risk of infection, low-dose aspirin could be prescribed, and care modifications may be individualized (4-6). However, the Royal College of Obstetricians and Gynaecologists (RCOG) guideline suggested aspirin for women with thrombocytopenia or platelets lower than 50000/mL should not be prescribed (7).

Some research suggests no evidence of acute side effects and long-term negative effects or a significant decrease in quality of life in pregnant women infected with COVID-19 taking NSAIDs (8). Moreover, according to mentioned guidelines, it seems that during the COVID-19 pandemic, it is necessary to screen the pregnant women in the first trimester for preeclampsia and offer low-dose aspirin for the ones who become high-risk. 
